# Open Cervical Approach for Zenker’s Diverticulum: A Retrospective Case Series

**DOI:** 10.1155/cris/1020020

**Published:** 2026-04-09

**Authors:** Beatriz Remezal Serrano, Juan Pérez Legaz, Monica Rey Riveiro, Pilar Serrano Paz

**Affiliations:** ^1^ Department of General and Digestive Surgery, Hospital Universitario del Vinalopó, Calle Tonico Sansano Mora, 14, 03293, Elche, Spain

**Keywords:** cervical surgery, cricopharyngeal myotomy, diverticulectomy, esophageal diverticulum, surgical outcomes, Zenker’s diverticulum

## Abstract

Zenker’s diverticulum (ZD) is an acquired outpouching of the mucosa and the submucosa through a point of weakness between the inferior pharyngeal constrictor and the cricopharyngeus muscles. It commonly presents with dysphagia, regurgitation, and halitosis. We retrospectively reviewed seven patients treated with open cervical surgery between 2010 and 2025. Six underwent diverticulectomy and cricopharyngeal myotomy, and one underwent diverticulopexy. The mean age was 59.4 years, and 85.7% were male. All patients experienced dysphagia, and regurgitation and halitosis were present in 42.8% each. Diagnosis was established through a barium swallow study, with complementary imaging performed in selected cases. No postoperative complications or mortality occurred. The mean hospital stay was 2.4 days, and after a mean follow‐up of 7 months, no recurrences or reinterventions were observed. Open cervical surgery remains a safe and effective option in properly selected patients, although the small sample size and short follow‐up warrant cautious interpretation of these findings.

## 1. Introduction

Zenker’s diverticulum (ZD) is a pulsion diverticulum resulting from herniation of the mucosal and submucosal layers through Killian’s triangle, an area of muscular weakness between the cricopharyngeus and the thyropharyngeus muscles [1]. This condition predominantly affects elderly males and is characterized by symptoms such as progressive oropharyngeal dysphagia, regurgitation of undigested food, chronic cough, halitosis, and, in severe cases, aspiration pneumonia [1, 2]. The pathophysiology involves impaired relaxation or hypertrophy of the upper esophageal sphincter, leading to increased intraluminal pressure and subsequent mucosal protrusion [1, 2].

Diagnosis is primarily established through a barium swallow study, which delineates the size and location of the diverticulum [1, 2]. Endoscopic evaluation and cervical computed tomography (CT) scans are adjunctive tools, particularly useful in assessing anatomical variations and planning surgical interventions [1].

Treatment modalities for ZD have evolved over the years and currently include three main therapeutic strategies: open transcervical surgery, rigid endoscopic stapling, and flexible endoscopic techniques [1, 3]. Open transcervical diverticulectomy with cricopharyngeal myotomy has traditionally been considered the reference approach because it provides direct visualization, complete sac management, and a formal myotomy [1, 3]. Rigid endoscopic stapling offers a less invasive transoral alternative in selected patients with adequate neck extension and diverticula large enough to allow proper exposure, although it may be less suitable for small pouches because the septum may not be divided completely [3–5]. Flexible endoscopic techniques, including septotomy and peroral endoscopic myotomy (Z‐POEM), have gained popularity due to their minimally invasive nature and favorable postoperative recovery profiles, particularly in small‐ to medium‐sized diverticula and in patients with higher surgical risk [4, 6, 7]. The choice of treatment should therefore be individualized according to diverticulum size, local anatomy, patient comorbidities, risk profile, and institutional expertise and availability of therapeutic endoscopy [1, 2, 4, 8, 9].

## 2. Case Presentation

**Figure 1:**
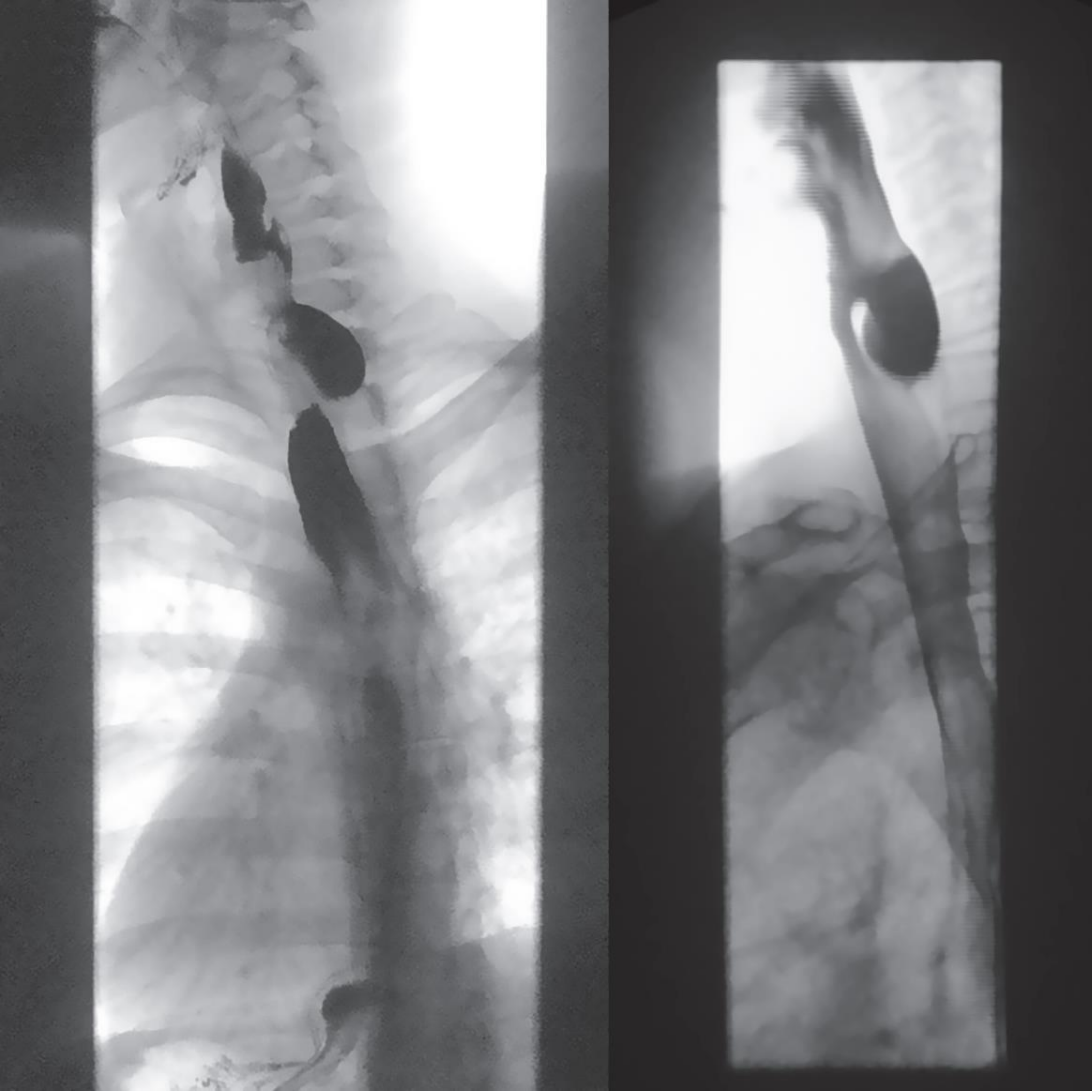
Contrast esophagogram study (composite images) showing a Zenker’s diverticulum arising posteriorly from the hypopharynx.

Seven patients diagnosed with ZD between 2010 and 2025 were reviewed. The mean age was 59.4 years (range 35–74), with a male predominance (85.7%). All patients reported dysphagia; regurgitation and halitosis were each present in 42.8% of cases, and vomiting was present in 28.5%. One patient (14.2%) had associated gastroesophageal reflux disease (GERD). Diagnosis was confirmed by barium swallow in all patients (Figure 1). Cervical CT was performed in three cases (42.8%) and upper gastrointestinal endoscopy in two cases (28.5%). Diverticulum size ranged from 2 to 5 cm, with three patients having diverticula measuring more than 3 cm (Table 1).

**Table 1:** Clinical, diagnostic, and surgical characteristics of patients treated for Zenker’s diverticulum.

Patient	Age (years)	Sex	Dysphagia	Regurgitation	Halitosis	Vomiting	GERD	Diagnosis by UGI series	Cervical CT	Diverticulum size (cm)	Surgical approach	Surgical technique	Postoperative complications	Recurrence	Reoperation
1	57	Male	Yes	No	No	Yes	Yes	Yes	No	4	Open cervical	Diverticulectomy + cricopharyngeal myotomy	No	No	No
2	74	Male	Yes	Yes	No	Yes	Yes	Yes	Yes	3	Open cervical	Diverticulectomy + cricopharyngeal myotomy	No	No	No
3	56	Male	Yes	Yes	Yes	No	Yes	Yes	No	2	Open cervical	Diverticulectomy + cricopharyngeal myotomy	No	No	No
4	64	Male	Yes	No	Yes	No	Yes	Yes	Yes	2	Open cervical	Diverticulectomy + cricopharyngeal myotomy	No	No	No
5	68	Male	Yes	No	No	No	Yes	Yes	Yes	4	Open cervical	Diverticulectomy + cricopharyngeal myotomy	No	No	No
6	35	Male	Yes	Yes	Yes	No	No	Yes	No	2	Open cervical	Diverticulectomy + cricopharyngeal myotomy	No	No	No
7	62	Male	Yes	Yes	Yes	No	No	Yes	No	5	Open cervical	Diverticulopexy	No	No	No

**Figure 2:**
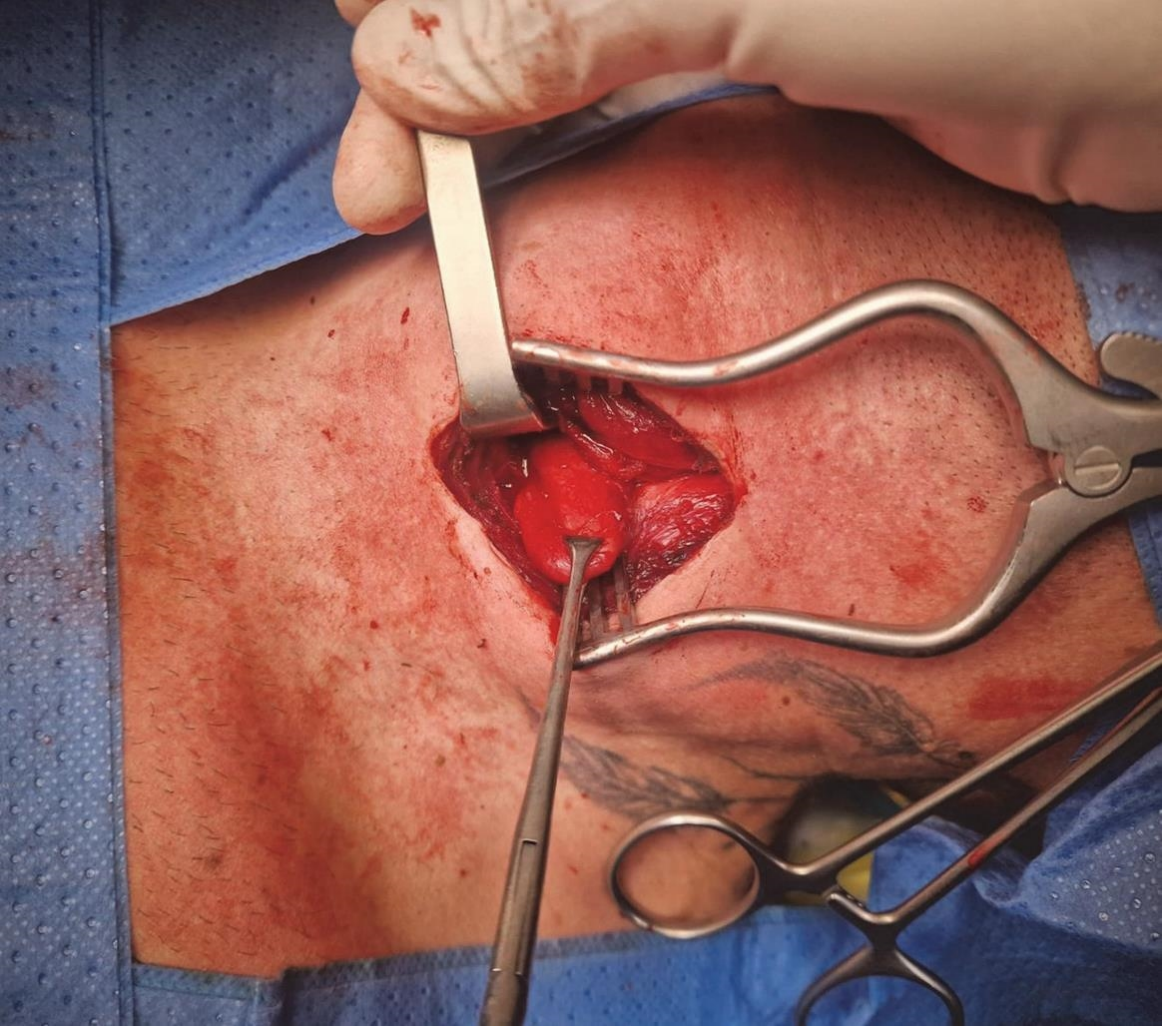
Intraoperative view showing a Zenker’s diverticulum fully dissected through a cervical incision, prior to resection using an endoscopic stapling device.

All patients underwent open cervical surgery: diverticulectomy and cricopharyngeal myotomy in six cases (85.7%) and diverticulopexy in one case (14.3%) (Figure 2). No intraoperative or postoperative complications, such as leakage, bleeding, infection, pneumonia, or need for reoperation, were observed. The mean hospital stay was 2.4 days (range 2–4 days). After a mean follow‐up of 7 months (range 2–12 months), no recurrences or need for reintervention was recorded.

## 3. Discussion

The management of ZD remains a subject of clinical debate, and the main current options can be grouped into three approaches: open cervical surgery, rigid endoscopic stapling, and flexible endoscopic therapy [1, 3, 4]. Presenting the outcomes of an open series is therefore most useful when placed within this therapeutic context and when the potential indications for each option are clearly stated.

Open transcervical surgery offers the advantage of direct exposure, complete treatment of the diverticular pouch, and a precise cricopharyngeal myotomy, which may translate into durable symptom relief and low recurrence rates [1, 3, 4]. This approach may still be particularly useful in patients with recurrent disease after previous treatment, in those with large diverticula or distorted cervical anatomy, and in situations where complete sac excision is preferred [1, 3, 4]. However, these benefits must be balanced against its greater invasiveness and the known risk of adverse events such as leaks, recurrent laryngeal nerve injury, wound complications, and longer postoperative recovery when compared with endoscopic alternatives [3, 8].

Rigid endoscopic stapling represents an established minimally invasive option, but its applicability depends on adequate transoral exposure and the availability of specific equipment [1, 3, 4]. In addition, this technique may be less suitable for small diverticula because the stapler may not divide the septum completely at its distal end, potentially leaving residual symptoms or favoring recurrence [3, 5]. Flexible endoscopic techniques, including septotomy and Z‐POEM, have expanded the therapeutic armamentarium for ZD and constitute a valuable alternative in centers with expertise in therapeutic endoscopy [1, 5–7]. These approaches are especially attractive in small to medium diverticula, in frail patients, and in those considered poor candidates for transcervical surgery because they avoid a cervical incision and usually allow faster recovery, although repeat intervention may sometimes be required and outcomes are closely linked to endoscopic expertise [1, 4–7, 9].

In this context, our case series supports the continued role of open cervical management in properly selected patients. In our institution, all patients were treated by an open approach, with no postoperative complications or recurrences during follow‐up. Although the sample is small, these findings suggest that open surgery remains a safe and effective option when recurrence, large pouch size, anatomical complexity, or local limitations in therapeutic endoscopy make a transcervical strategy more appropriate. In our series, diverticulum size ranged from 2 to 5 cm, and three patients had diverticula measuring more than 3 cm, a feature that may also have supported the choice of an open approach in selected cases. Rather than demonstrating only that the procedure can be performed, our experience highlights the clinical niche in which open surgery may still offer relevant advantages.

The main limitations of this study are its retrospective design, the small sample size, and the relatively short follow‐up. However, ZD is an uncommon condition, and surgical treatment is usually reserved for selected patients, which may explain the limited number of cases collected over a prolonged study period. Although our results suggest that open cervical surgery remains a safe and effective option in appropriately selected patients, the mean follow‐up of 7 months (range 2–12 months) is insufficient to adequately assess the long‐term durability of symptom relief and recurrence, which may occur years after surgery. Accordingly, our favorable short‐term outcomes should be interpreted with caution and would benefit from confirmation in larger series with longer follow‐up [1, 3, 4, 8].

## Funding

This research received no external funding.

## Conflicts of Interest

The authors declare no conflicts of interest.

## Data Availability

The data that support the findings of this study are available from the corresponding author upon reasonable request, subject to privacy and ethical restrictions.

## References

[1] Ramamurthy S. , Ahuja P. , and Dahiya D. S. , et al.Management Strategies for Zenker’s Diverticulum: A Comprehensive Review, Journal of Clinical Medicine. (2025) 14, no. 17, 10.3390/jcm14176141, 6141. 40943901 PMC12429033

[2] Fair L. and Ward M. A. , Modern Approaches to Treating Zenker’s Diverticulum, Current Opinion in Gastroenterology. (2023) 39, no. 4, 333–339, 10.1097/MOG.0000000000000941. 37278290

[3] Bhatt N. K. , Mendoza J. , Kallogjeri D. , Hardi A. C. , and Bradley J. P. , Comparison of Surgical Treatments for Zenker Diverticulum: A Systematic Review and Network Meta-Analysis, JAMA Otolaryngology–Head & Neck Surgery. (2021) 147, no. 2, 190–196, 10.1001/jamaoto.2020.4091. 33270099 PMC7716255

[4] Rudler F. , Pineton de Chambrun G. , and Lallemant B. , et al.Management of the Zenker Diverticulum: Multicenter Retrospective Comparative Study of Open Surgery and Rigid Endoscopy Versus Flexible Endoscopy, Surgical Endoscopy and Other Interventional Techniques. (2023) 37, no. 9, 7064–7072, 10.1007/s00464-023-10225-4. 37380740

[5] Kaminski M. F. , Budnicka A. , Przybysz A. , and Pilonis N. D. , Traditional Septotomy or Z-POEM for Zenker’s Diverticulum, Best Practice & Research Clinical Gastroenterology. (2024) 71, 10.1016/j.bpg.2024.101943, 101943. 39209416

[6] Repici A. , Spadaccini M. , and Belletrutti P. J. , et al.Peroral Endoscopic Septotomy for Short-Septum Zenker’s Diverticulum, Endoscopy. (2020) 52, no. 7, 563–568, 10.1055/a-1127-3304. 32185781

[7] Hernández Mondragón O. V. , Solórzano Pineda M. O. , and Blancas Valencia J. M. , Zenker’s Diverticulum: Submucosal Tunneling Endoscopic Septum Division (Z-POEM), Digestive Endoscopy. (2018) 30, no. 1, 124–124, 10.1111/den.12958, 2-s2.0-85041464950. 28875504

[8] DeKloe J. , Shing S. R. , Herzig L. , Bertoni D. , and Tibbetts K. M. , Complications Following Surgical Management of Zenker Diverticulum: A Comparative Analysis of Endoscopic and Open Approaches, Otolaryngol Head Neck Surg. (2025) 172, no. 4, 1328–1333, 10.1002/ohn.1119. 39764670

[9] Holland A. M. , Lorenz W. R. , Ricker A. B. , Mead B. S. , Scarola G. T. , and Colavita P. D. , Cricopharyngomyotomy: Outcomes of Flexible Endoscopic Management of Small and Medium Sized Zenker’s Diverticulum, The American Journal of Surgery. (2024) 238, 10.1016/j.amjsurg.2024.115823, 115823. 38981838

